# Geostatistical Modeling of Malaria Endemicity using Serological Indicators of Exposure Collected through School Surveys

**DOI:** 10.4269/ajtmh.14-0620

**Published:** 2015-07-08

**Authors:** Ruth A. Ashton, Takele Kefyalew, Alison Rand, Heven Sime, Ashenafi Assefa, Addis Mekasha, Wasihun Edosa, Gezahegn Tesfaye, Jorge Cano, Hiwot Teka, Richard Reithinger, Rachel L. Pullan, Chris J. Drakeley, Simon J. Brooker

**Affiliations:** Malaria Consortium, London, United Kingdom; Faculty of Infectious and Tropical Diseases, London School of Hygiene and Tropical Medicine, London, United Kingdom; Malaria Consortium, Addis Ababa, Ethiopia; Ethiopian Public Health Institute, Addis Ababa, Ethiopia; Oromia Regional Health Bureau, Addis Ababa, Ethiopia; President's Malaria Initiative, United States Agency for International Development, Addis Ababa, Ethiopia; RTI International, Washington, District of Columbia

## Abstract

Ethiopia has a diverse ecology and geography resulting in spatial and temporal variation in malaria transmission. Evidence-based strategies are thus needed to monitor transmission intensity and target interventions. A purposive selection of dried blood spots collected during cross-sectional school-based surveys in Oromia Regional State, Ethiopia, were tested for presence of antibodies against *Plasmodium falciparum* and *P. vivax* antigens. Spatially explicit binomial models of seroprevalence were created for each species using a Bayesian framework, and used to predict seroprevalence at 5 km resolution across Oromia. School seroprevalence showed a wider prevalence range than microscopy for both *P. falciparum* (0–50% versus 0–12.7%) and *P. vivax* (0–53.7% versus 0–4.5%), respectively. The *P. falciparum* model incorporated environmental predictors and spatial random effects, while *P. vivax* seroprevalence first-order trends were not adequately explained by environmental variables, and a spatial smoothing model was developed. This is the first demonstration of serological indicators being used to detect large-scale heterogeneity in malaria transmission using samples from cross-sectional school-based surveys. The findings support the incorporation of serological indicators into periodic large-scale surveillance such as Malaria Indicator Surveys, and with particular utility for low transmission and elimination settings.

## Introduction

With the rekindling of malaria elimination goals,^1^,^2^ there is an increased need to quantify patterns of and changes in malaria risk to support evidence-based targeting of interventions, implement surveillance strategies to monitor changes in transmission intensity, and assess feasibility of local elimination.^3^,^4^

Low-transmission settings present specific challenges to implementation of cross-sectional surveys: 1) highly seasonal transmission can result in underestimates of population parasite rates if sampling does not occur during the peak transmission period; 2) low-density infections are frequent and either will be underestimated by microscopy or rapid diagnostic tests (RDTs),^5^,^6^ or require significantly increased resources to screen samples by polymerase chain reaction (PCR); and 3) where diagnostic data are used to develop spatial prediction models, there is a risk that the true extent of transmission will be underestimated since recently cleared and low-density infections will not be included. While new strategies such as reactive case detection^7^–^9^ and “rolling” cross-sectional surveys^10^ have been trialed, there remains a need to develop strategies to track changes in low and unstable transmission settings.

Detection of anti-*Plasmodium* antibodies eluted from dried blood spots has been demonstrated to be robust,^11^,^12^ yielding estimates of seroprevalence and seroconversion rates that are representative of malaria transmission intensity within a community.^13^,^14^ Because antibodies persist after infection clearance, they offer the opportunity to examine exposure to malaria over a wider period than is typically possible through detection of parasitemia during a cross-sectional survey by means of microscopy, RDTs, or PCR-based methods.

Serological indicators are increasingly being used in community-based malaria epidemiological studies to assess small- and large-scale spatial heterogeneities of and changes in transmission.^13^,^15^–^19^ Schools provide a useful alternative platform for collection and monitoring of malariometric indicators, offering logistical advantages (e.g., simplified selection of participants, high compliance, and reduced survey costs) over standard community-based cross-sectional surveys.^20^–^22^ Although schools consistently yield higher estimates, school-survey seroprevalence estimates have repeatedly been shown to strongly correlate with community-survey seroprevalence.^23^

This study explored the use of serological indicators collected from a large-scale school-based survey to describe differences in *P. falciparum* and *P. vivax* endemicity in a low-transmission setting. Spatially explicit Bayesian modeling techniques were used to explore relationships between serological indicators at population level and explanatory environmental variables, to predict estimated endemicity levels at subnational scale.

## Methods

### Study setting.

Ethiopia has a diverse ecology, and malaria transmission is known to be spatially heterogeneous, related to variables such as altitude, temperature, rainfall, and presence of local water bodies or dams.^24^–^28^ Malaria transmission is temporally variable because of seasonal rainfall, with a major transmission season from September to December and a minor transmission season from April to May. Cases are due to both *P. falciparum* and *P. vivax*. The Malaria Indicator Survey in 2011 demonstrated a low parasite prevalence within the population living in malaria-risk areas, estimated at 1.3% by microscopy and 4.5% by RDT in areas < 2,000 m.^29^

### Survey data.

Data presented in this paper are drawn from a large cross-sectional survey conducted in 197 government primary schools in Oromia Regional State, Ethiopia, in 2009.^30^ Full detail of school and child selection as well as sample collection has been presented elsewhere.^30^ In brief, at each school 55 girls and 55 boys were randomly selected. They provided finger-prick blood samples for preparation of thick- and thin-blood smears, hemoglobin measurement (HemoCue Ltd., Angelhölm, Sweden), and collection of blood spots on filter paper (Whatman 3MM; Whatman, Maidstone, United Kingdom). School location was measured using a global positioning satellite receiver (eTREX; Garmin International, Olathe, KS).

For serological analysis samples were selected purposively from 1) 20 schools with highest prevalence of *Plasmodium* infection detected by microscopy (range 0.9–14.5%); 2) 20 schools with highest proportion of anemic (classified according to WHO,^31^ including adjustment by altitude) children (range 34.2–51.4%); and 3) a random selection of remaining schools surveyed (Table 1). Purposive selection was conducted to capture a range of transmission settings, and since resources were not available to complete enzyme-linked immunosorbent assay (ELISA) on all blood spots collected during surveys.

### Enzyme-linked immunosorbent assay.

Blood spots from 50 schools were analyzed in London, United Kingdom, against *P. falciparum* merozoite surface protein-1_19_ (*Pf*MSP-1), *P. falciparum* glutamate-rich protein-R2 (*Pf*GLURP), *P. vivax* MSP 1_19_ (*Pv*MSP-1), and *P. vivax* apical membrane antigen-1 (*Pv*AMA). In Addis Ababa, Ethiopia, blood spots from a further 12 schools were analyzed against *Pf*MSP-1 and 21 schools against *Pv*MSP-1.

Antibodies were eluted from dried blood spots, and samples were tested for IgG against *P. falciparum* and *P. vivax* antigens according to methods described elsewhere.^12^ Duplicate optical density (OD) values with > 20% variation were excluded. Raw ODs were corrected by blank OD and normalized between plates by fitting to the midpoint of a standard curve produced by serial dilution of hyperimmune serum (i.e., pooled hyperimmune serum from Tanzania for *P. falciparum*, and reconstituted *P. vivax* and *P. malariae* hyperimmune serum (NIBSC code 72/096, Hertforshire, UK) for *P. vivax*). Normalized ODs and identification numbers were exported into Stata 12.0 (STATA Corporation, College Station, TX). Individual samples were classified as seropositive or seronegative against each antigen using a mixture model, whereby the mean of the seronegative distribution plus three standard deviations was defined as the seropositive cutoff.^12^,^13^ Binary variables were generated to describe, in summary, seropositivity by species: for example, *P. falciparum* seropositive samples were defined as seropositive against either *Pf*MSP-1 and/or *Pf*GLURP. In the absence of a gold standard for anti-*Plasmodium* antibody detection, it is not possible to determine the sensitivity or specificity of the ELISA, but the mixture model approach is commonly used in low-transmission settings,^15^,^32^–^34^ where the population is expected to include true seronegative and true seropositive individuals. Various nonlinear regression functions were fitted to scatter plots of microscopy prevalence and seroprevalence for each species separately using least squares regression, to describe the relationship between microscopy and serology.

### Remote sensing environmental data.

Elevation data were extracted from the Shuttle Radar Topography Mission (SRTM) digital elevation model at 90 m resolution,^35^ resampled to 250 m, and further processed to estimate slope in degrees. Gridded precipitation and temperature data at 1 km resolution were extracted from preprocessed data available on WorldClim.^36^,^37^ This source provides a set of data layers generated through interpolation of average monthly climate data obtained during the period 1950–2000. Euclidean distance to water bodies was calculated using SRTM Water Body Data files at 250 m resolution,^38^ and distance to rivers and roads calculated using data from Digital Chart of the World at 250 m resolution.^39^ Land cover type was extracted from the qualitative global land cover map for 2005, defined within the United Nations land cover classification system using environmental satellite (ENVISAT) mission's Medium Resolution Imaging Spectrometer (MERIS) sensor at 300 m resolution.^40^ Monthly raster datasets of normalized difference vegetation index (NDVI) indicators at 1 km resolution were extracted from the SPOT 5 vegetation project^41^ for the period 2005 and 2009. Gridded mean, minimum, maximum, and standard deviations were obtained for the entire period and for the specific survey year (2009). Population density was extracted from the AfriPop project at 100 m resolution,^42^ and rural–urban classification at 1 km from the Global Rural-Urban Mapping Project (GRUMP).^43^ Input grids were either extended or clipped to match the geographic extent of a land mask template, aligned to it, and eventually resampled to a 5 km resolution by bilinear interpolation for continuous surface, and majority approach for categorical data. Bilinear interpolation determines the new value of a cell based on a weighted distance average of the nearest input cell centers, whereas majority approach determines the new value based on the most popular values within the resampling window. We assumed that both methods cause some smoothing of the data. Environmental data were extracted to school locations using ArcMap 12.0 (Environmental Systems Research Institute Inc., Redlands, CA).

### Model development and testing.

Environmental and serology data were merged and analyzed using Stata 12.0. Continuous environmental variables were standardized to facilitate later model convergence. Models were developed separately to describe *P. falciparum* or *P. vivax* seroprevalence.

Univariate associations between school seroprevalence and environmental variables were explored, and colinearity (correlation coefficient > 0.9) between variables was tested. A school-level minimal adequate logistic regression model was developed by the backward stepwise method, whereby variables with *P* > 0.05 were removed in the order of least significance; all excluded variables were subsequently retested in the final model. Akaike information criterion (AIC) and Bayesian information criterion (BIC) were used to inform model selection.^44^,^45^

Four multivariate Bayesian binomial regression models were developed using WinBUGS 1.4 (Medical Research Council Biostatistics Unit and Imperial College London, London, United Kingdom) for *P. falciparum* and for *P. vivax.* The most complex model included the retained school-level environmental variables, school-level random effect, and school-level geostatistical random effect (using an isotropic, stationary exponential decay function).^46^ Additional models excluded the environmental variables, the spatial random effect, or both.

Semi-informative priors were set for the rate of decay of spatial correlation, *φ*, informed by the maximum and minimum distance between schools, and non-informative priors used for other coefficients. Models were burned in for 10,000 iterations to achieve convergence, and then nodes were sampled for 10,000 iterations, thinning each 10 iterations. Final model selection was informed by examining the variance of school and spatial random effects and deviance information criterion (DIC).^47^

### Model validation.

Models were externally validated by training the model on an N-5 school dataset, then predicting probability that seroprevalence thresholds (2%, 5%, and 40%) exceeded for the five excluded schools. These thresholds were estimated to describe the lowest and highest areas of seroprevalence, and hence endemicity, to support specific intervention targeting. The process was repeated until predictions for all schools were available. Model performance was assessed by examining the area under the curve (AUC) of the receiver operator characteristic (ROC) at each threshold.^48^ AUC > 0.7 indicates a reasonable discriminative capacity, and AUC > 0.9 very good discriminative capacity.^49^,^50^

### Generating a predictive seroprevalence map.

A grid of 12,048 locations at 5 km spacing was generated across Oromia, and environmental variables included in final models were extracted to these locations. The selected Bayesian models were trained on actual school seroprevalence data, then predicted at each location by calculating the sum of the products of the covariate coefficients and the values of the covariates at each grid node, plus the interpolated geostatistical random effect, and back transforming from the logit to the prevalence scale.

### Ethical considerations.

The school surveys received ethical clearance from the Ethiopian Science and Technology Agency (RDHE/2-89/2009), with additional clearance subsequently given for serological analysis of blood spots (3.10/53/2003). Consent for participation used a passive, opt-out procedure, with school director providing written consent for the survey to proceed. Schools were requested to hold meetings in advance with parents to inform them of the survey and allow withdrawal of children if necessary. Participating children gave written assent and were informed of their right to withdraw at any time. Children reporting fever during surveys were tested with a multispecies HRP2-panLDH RDT (CareStart; AccessBio, Somerset, NJ), and any child with a positive RDT was treated according to the national guidelines.^51^

## Results

### Serology findings.

Serology results were available for *P. falciparum* from 5,914 children from 62 schools, with a mean 95 (range 10–111) samples per school. *Plasmodium vivax* results were available from 6,609 children from 71 schools, with mean 93 (range 5–111) samples per school. Data were from children aged 5 to 18 years (mean 11 years).

Of all children tested, 11.6% (688/5,913) were *P. falciparum* seropositive and 11.1% (735/6,609) *P. vivax* seropositive; 1.0% and 0.5% children were microscopy-positive for *P. falciparum* and *P. vivax* parasites, respectively. Cross-tabulation of microscopy and antigen-specific serology results are presented in Table 2. Where data were available for both species, 4.7% of 5,420 children were seropositive against both species. When restricting our analyses to schools with more than 50 children tested (56 schools for *P. falciparum*, 62 for *P. vivax*), *P. falciparum* and *P. vivax* school seroprevalence ranged from 0% to 50% and 0% to 53.7%, respectively.

Among 50 schools tested against 4 antigens, correlation was seen between school seroprevalence determined for *Pf*MSP-1 and *Pf*GLURP (*R*^2^ = 0.84), and for *Pv*MSP-1 and *Pv*AMA (*R*^2^ = 0.80). For both species, coating plates with MSP-1 resulted in higher sensitivity than *Pf*GLURP or *Pv*AMA. A strong correlation (*R*^2^ = 0.84) was seen between school *P. falciparum* and *P. vivax* seroprevalence.

### Comparing serology to microscopy.

**Figure 1.**
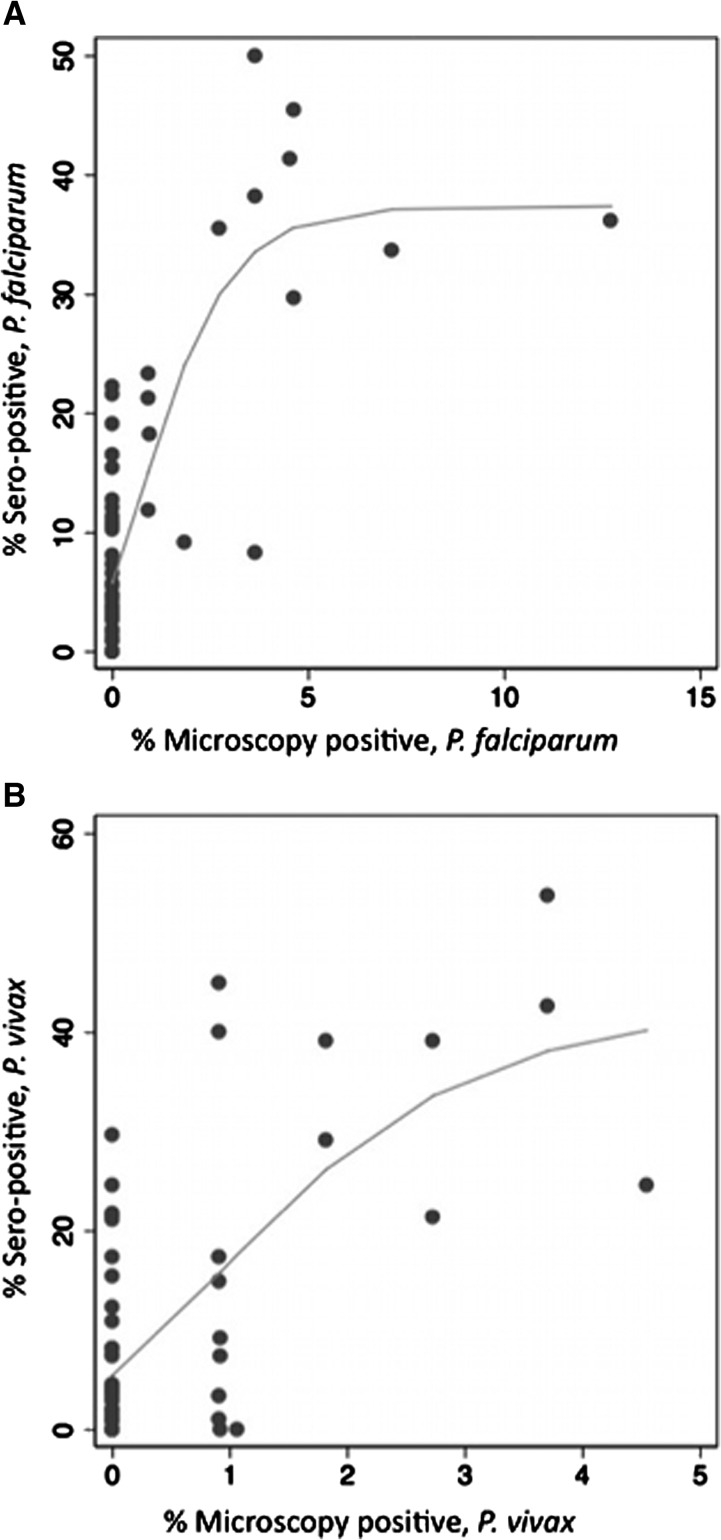
School-level seroprevalence and prevalence of infection detected by microscopy for *Plasmodium falciparum* (**A**) and *P. vivax* (**B**). Scatter plots are presented for 56 schools with *P. falciparum* data and 62 schools with *P. vivax* data, restricted to those with serology results from ≥ 50 children. Nonlinear regression identified a Gompertz function as best fit to *P. falciparum* (*R*^2^ = 0.810), and to *P. vivax* data (*R*^2^ = 0.657).

Schools with 0% positive samples by microscopy were found to have from 0% to 30% seroprevalence. Although the proportion of microscopy positive and seropositive children in a school are not directly comparable, it is plausible to expect some association between the two measures (Figure 1).

### Environmental risk factors.

Colinearity was found among the precipitation, temperature and NDVI variables, and between distance to both permanent and any type of water body. Distance to water bodies and rivers, land gradient, and urban areas showed univariate associations with both *P. falciparum* and *P. vivax* seroprevalence (Supplemental Table 1).

The minimal adequate multivariate *P. falciparum* frequentist model includes elevation and angle of land slope, distance to permanent river, bare or sparse land cover, population density, and urban areas. The minimal adequate *P. vivax* frequentist model includes distance to permanent river and water body, precipitation during the wettest quarter of the year (projection from 1950 to 2000 to allow for high spatial resolution), and mean NDVI over the preceding 5 years.

### Bayesian modeling of *P. falciparum*.

When comparing output from nonspatial and spatial models of *P. falciparum*, incorporating spatial structure in models was found to explain much of the variation between schools, indicated by a reduction of *σ*^2^*_school_* when spatial random effects were included in models. A lower DIC in models including spatial structure justified retention in the final *P. falciparum* Bayesian model.

**Figure 2.**
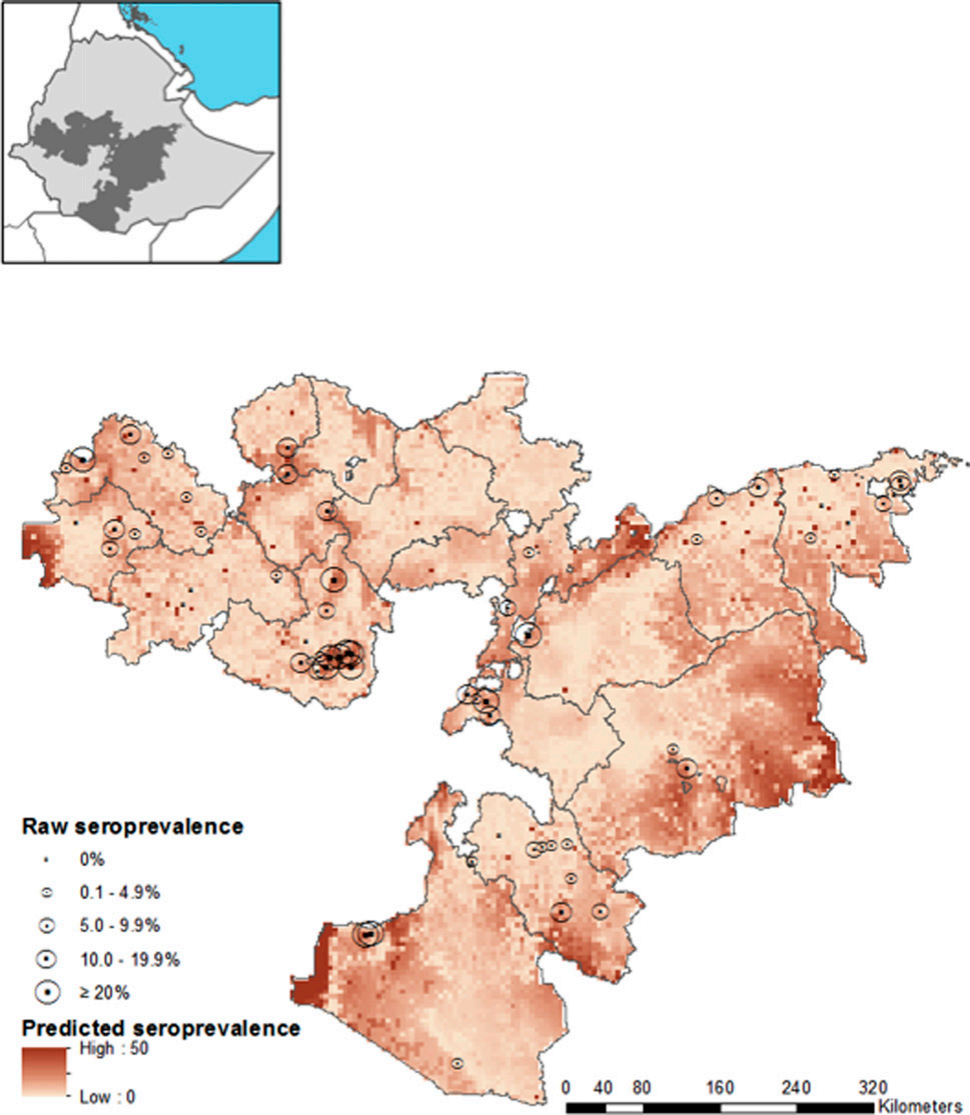
Map of predictive *Plasmodium falciparum* seropositivity using spatial model with environmental fixed effects. Measured *P. falciparum* seroprevalence from the 62 schools used to train the model are shown by circles with size proportional to seroprevalence. Inset map indicates the location of Oromia Regional State (shaded) within Ethiopia.

**Figure 3.**
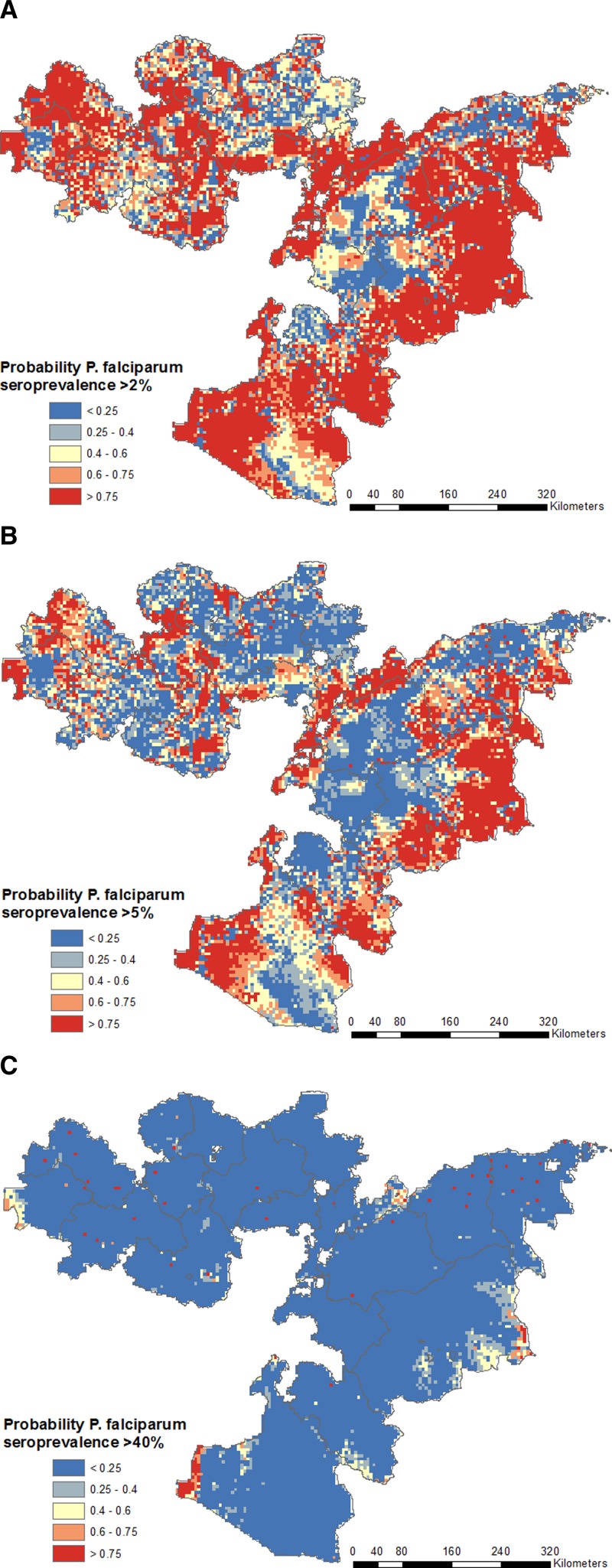
Probability of *Plasmodium falciparum* seroprevalence exceeds the defined thresholds of 2% (**A**), 5% (**B**), and 40% (**C**) according to final predictive model for *P. falciparum*. Red areas are those very likely to exceed the threshold, blue areas very unlikely to exceed the threshold, and pale yellow areas have high uncertainty.

Inclusion of environmental variables in the spatial model was shown to reduce *σ*^2^*_spatial_* and the DIC, as well as increase the rate of decay of spatial correlation (ϕ), indicating that much of the first-order spatial variation can be explained adequately by the included environmental data. Therefore, the final model to describe *P. falciparum* seroprevalence in Oromia incorporates environmental covariates to explain first-order deterministic spatial variation, with the spatial random effect adequately capturing second-order structure (Table 3). The final model had mean prediction error of −0.31, indicating a tendency to moderately underpredict *P. falciparum* seroprevalence. Mean absolute error of the model, indicating the magnitude of error in predictions, was 6.64. Internal validation demonstrates a good discriminatory ability of the final model for 2% and 5% seroprevalence thresholds, with an AUC of 0.83 and 0.84, respectively. The model performs very well in identifying areas of over 40% seroprevalence (AUC = 0.96). Actual and predicted school seroprevalence were found to be correlated (Pearson *r* = 0.62, *P* < 0.001). The final model was used to predict *P. falciparum* seroprevalence at 5 km resolution across Oromia, the posterior mean is shown in Figure 2

, with probability of 2%, 5%, and 40% thresholds shown in Figure 3

.

### Bayesian modeling of *P. vivax*.

**Figure 4.**
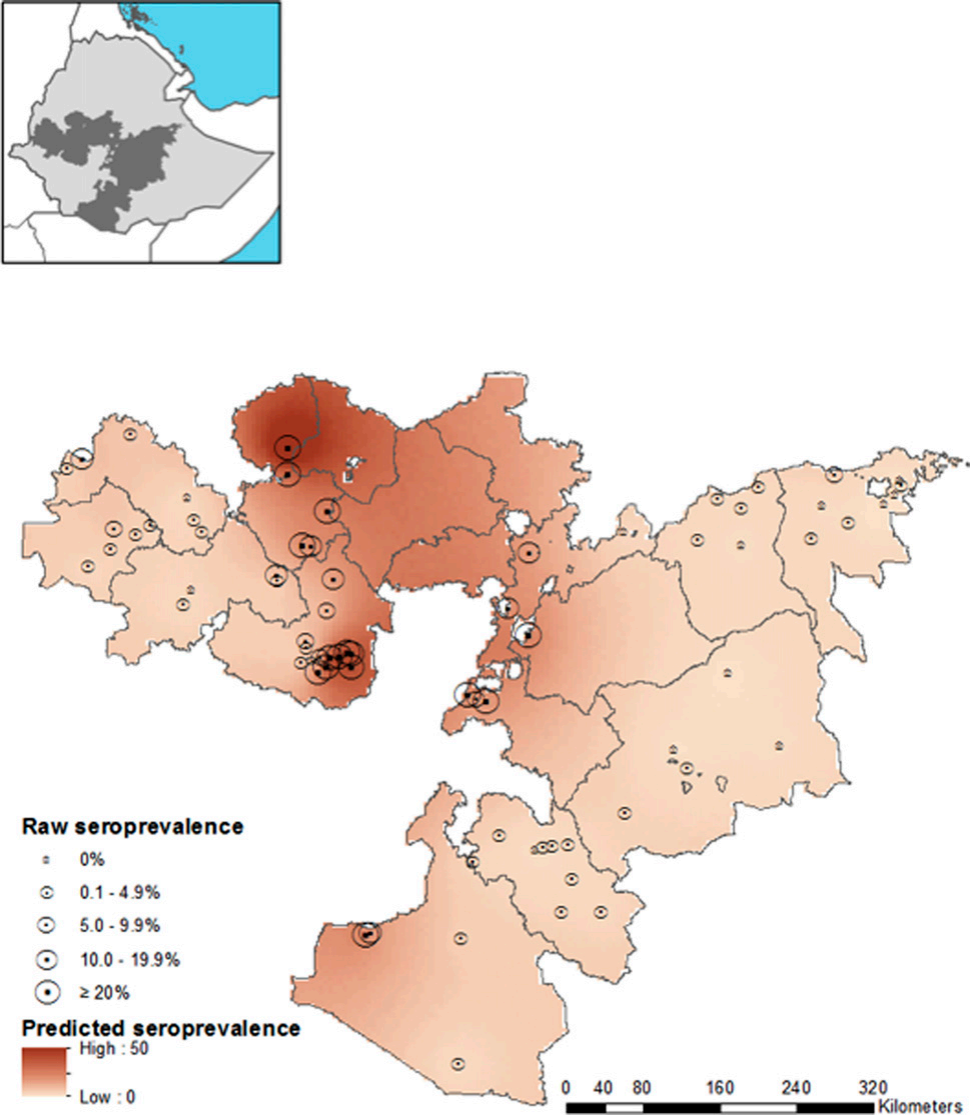
Map of predictive *Plasmodium vivax* seropositivity, using spatial model without environmental fixed effects. Measured *P. vivax* seroprevalence from the 71 schools used to train the model are shown by circles with size proportional to seroprevalence. Inset map indicates the location of Oromia Regional State (shaded) within Ethiopia.

**Figure 5.**
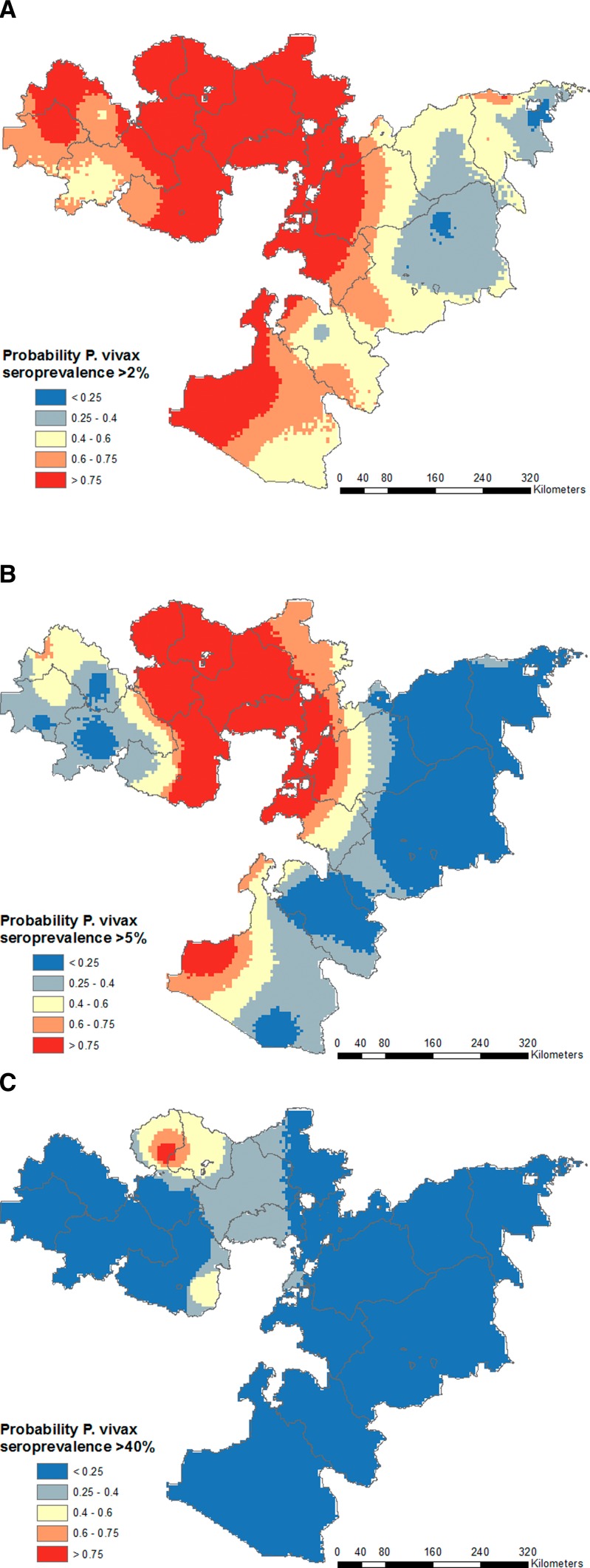
Probability of *Plasmodium vivax* seroprevalence exceeds the defined thresholds of 2% (**A**), 5% (**B**), and 40% (**C**) according to final predictive model for *P. vivax*. Red areas are those very likely to exceed the threshold, blue areas very unlikely to exceed the threshold, and pale yellow areas have high uncertainty.

Similar to the *P. falciparum* models, incorporating spatial structure in *P. vivax* models was found to explain much of the variation between schools, indicated by a reduction of *σ*^2^*_school_* and lower DIC. Inclusion of environmental variables in the spatial *P. vivax* model did not substantially reduce the *σ*^2^*_spatial_*, and little difference was seen in ϕ and DIC between the models with and without environmental variables. The final model for *P. vivax* is, therefore, the spatial model with no environmental covariates (Table 3). The final model had mean prediction error of 0.03, and mean absolute error, indicating the magnitude of error in predictions, of 6.64. Internal validation of this model indicates good performance at the 2% seroprevalence threshold (AUC = 0.81) and very good performance at 5% and 40% thresholds (AUC = 0.91 for both). Actual and predicted seroprevalence were correlated (Pearson *r* = 0.68, *P* < 0.001). Predictions of the final model at 5 km resolution are displayed in Figure 4

as the posterior mean seroprevalence, and in Figure 5

as the probability of seroprevalence thresholds being exceeded.

## Discussion

This study shows the capability of serological markers to detect large-scale heterogeneity in malaria transmission using samples collected during cross-sectional school surveys, in a setting with seasonal and low transmission. Seroprevalence was found to be associated with environmental variables; this relationship was used to predict seroprevalence at unsampled locations using Bayesian geostatistical modeling methods incorporating fixed and random effects.

School seroprevalence determined by different antigens showed strong correlation for each species, with MSP showing higher sensitivity for both *P. falciparum* and *P. vivax* than *P. falciparum* GLURP and *P. vivax* AMA, respectively. However, previous studies have shown AMA-1 to have higher immunogenicity than MSP-1.^11^ As transmission declines, individual antibody responses become more disparate, and therefore it is recommended that future serological analysis be conducted using multiple antigens or a whole parasite lysate.

The range of seroprevalence found across schools where no children were microscopy positive during the cross-sectional survey demonstrates the value of serological indicators, that is, in differentiating between schools where transmission occurs but the peak transmission period was missed by surveys, and those with very low malaria risk. Differences may also be apparent if transmission has ceased in the area in recent years before the age of the youngest school child. This is difficult to demonstrate without clinical data.

Both species' final models incorporated a spatial random effect to describe spatial autocorrelation, whereby schools located closely together were more similar than schools at greater distance. All models included a nonspatial random effect. The *P. falciparum* model indicated that spatial autocorrelation was present to a distance of approximately 46 km, while the *P. vivax* model showed a range of over 500 km. The *P. falciparum* range is a distance at which similarities in climatic factors and ecology would be expected, and therefore it is feasible that these areas experience similar transmission intensity. However, the very large spatial range of *P. vivax* suggests that the spatial random effect is capturing other large-scale variations not tested for inclusion in the Bayesian spatial model. A similar finding was reported in spatial modeling of malaria in Bangladesh, where environmental variables described a large proportion of spatial variation in *P. falciparum*, but little of the *P. vivax* distribution.^52^

Frequentist models developed for *P. vivax* suggested biologically plausible environmental risk factors of distance to rivers and water bodies, vegetation cover, and precipitation; nonetheless, these did not adequately explain the large-scale trends in *P. vivax* seropositivity after accounting for spatial dependency. The final *P. vivax* map presented here therefore simply uses spatial interpolation to predict seroprevalence at unsampled locations. The larger spatial scale of *P. vivax* may be due in part to the production of hypnozoites, since the reactivation of parasites and subsequent antibody production may occur in a different location to site of parasite acquisition, or in the absence of ongoing transmission. However, it is unlikely that recrudescent infections would have had a major confounding effect on school seroprevalence, unless large-scale population movements would have occurred. Furthermore, the wider *P. vivax* range may be due to the parasite's ability to generate sporozoites at lower temperatures and the potential to be transmitted at higher altitudes.^53^ Indicators of temperature and altitude, considered to define vector survival and sporogeny, were not found to be associated with *P. vivax* seroprevalence, and not retained in the final multivariate model.

The key environmental variables identified for inclusion in the *P. falciparum* map indicated that higher risk exists in low-altitude and low-gradient areas close to rivers. We postulate that seasonal flooding in flatlands where floodwaters may pool and act as vector breeding sites could be the driver of this relationship.

A further extension to the current models in the future could be incorporation of intervention coverage, such as districts targeted by indoor residual spraying of households with insecticide, and estimations of long-lasting mosquito net coverage and use alongside environmental covariates. Furthermore, a Bayesian approach to selection of environmental covariates may have resulted in a different panel of covariates tested in final models.

Despite the difficulties in modeling *P. vivax* seroprevalence, our maps of both *P. falciparum* and *P. vivax* seroprevalence do show broad concordance with predictive maps developed by the Malaria Atlas Project to describe age-standardized parasite rates using model-based geostatistical prediction methods,^48^,^54^,^55^ with similar areas of Oromia identified as areas of highest and lowest risk for malaria. Survey locations with microscopy-positive samples in the most recent Ethiopian Malaria Indicator Survey in 2011 also correlate with our predictive map, with infections identified along the Rift Valley as well as in the far west of Oromia.^29^ Considering that sites included in the current modeling approach were selected using a combination of purposive and random methods, a further improvement to the approach presented here would involve defining a likelihood function more appropriate for preferentially sampled geostatistical data, as described in Diggle and others.^56^ The use of conventional geostatistical methods to model these data, which assumes non-preferential sampling, could potentially result in misleading inferences. Nevertheless, since a stratified random sampling approach was used for selection of the 200 schools included in the wider malariometric survey, and the use of both purposive and random methods to identify schools for serological testing, these results remain meaningful, even if a cautious interpretation considering potential biases is required.

This study was designed to evaluate large-scale spatial heterogeneity of *P. falciparum* and *P. vivax* malaria. Although logistical constraints limited the number of samples analyzed, the original surveys were powered to microscopy-based parasite rate—therefore, seroprevalence rates being higher than microscopy should mean that adequate samples were examined to evaluate associations with environmental variables and build the statistical model. The study was not designed to assess microheterogeneity in transmission within communities, which has been demonstrated in other settings with similarly low transmission levels (e.g., Somalia, The Gambia, Guinea-Bissau).^15^,^17^ The randomization process and use of school-attending children as a sampling frame should result in a sampled population representative of the whole school catchment area and wider community. We acknowledge that there is potential for school catchment areas in Ethiopia to have diversity in transmission intensity as a result of steep gradients and presence of local water bodies, dams, and irrigation systems.^24^,^26^ Individual differences in immune status and antibody production in response to *Plasmodium* antigen exposure are expected, and may be moderated by other parasitic infections, including helminthes^57^; yet, infection risks for these are likely to be broadly similar across all sites, and individual differences in immune response are likely to be randomly dispersed among the population.

The Bayesian spatially explicit models developed in this study could be refined by inclusion of serology data from additional sites, both within Oromia to assist in categorizing areas of high model uncertainty, as well as from other regional states to assist in developing a nationally representative risk map. Serological analysis of filter paper blood spots included in periodic national surveys such as Malaria Indicator Surveys or Demographic and Health Surveys would be a simple strategy to collect additional seroprevalence data nationally.

Generation of estimates of cluster seroprevalence can therefore complement the collection of parasitological indicators from periodic large scale surveys in low-transmission settings by indicating recent and historical transmission intensity, depending on population tested and antigens used. In settings with unstable transmission, these data may indicate receptivity to transmission,^58^,^59^ therefore can support policy makers in targeting interventions to areas of current transmission or at risk of transmission. The geostatistical map presents estimates of these serological indicators beyond the sampled locations, allowing evidence-based intervention targeting to take place beyond sampled clusters, along with estimates of model uncertainty demonstrating settings where further data may be needed for decision making.^60^,^61^

Should serology become a primary indicator for malaria surveillance, it may be worthwhile to review the recommended sampling strategy for serological indicators, to ensure a cost-efficient, timely and appropriately powered survey. Further developments to this work and exploration of the use of serological indicators as part of a package of surveillance tools in Ethiopia could be validation of measured seroprevalence and model predictions against other available data, including clinical burden recorded routinely at health facilities and cluster-level Malaria Indicator Survey data.

These data represent the spatial integration of simple survey design with a relatively basic laboratory assay that can subsequently guide malaria control and surveillance. The approach has particular utility in low-transmission settings and, therefore, has important applications for malaria elimination.

## Supplementary Material

Supplemental Table.

## Figures and Tables

**Table 1:** Number of schools and children tested by ELISA against each antigen, stratified by school selection criteria: high microscopy prevalence, high anemia prevalence, and randomly selected

	Any *P. falciparum* antigen				Any *P. vivax* antigen			
	Schools		Children		Schools		Children	
Total tested	62		5,913		71		6,609	
School selection criteria	*Pf*MSP-1		*Pf*GLURP		*Pv*MSP-1		*Pv*AMA	
	Schools	Children	Schools	Children	Schools	Children	Schools	Children
High microscopy prevalence	20	2,088	20	2,093	20	2,080	20	2,074
High anemia prevalence	20	2,118	20	2,092	20	2,080	20	2,104
Random selection	22	1,614	10	1,037	31	2,327	10	1,024
Total tested	62	5,820	50	5,222	71	6,487	50	5,202

AMA = apical membrane antigen; ELISA = enzyme-linked immunosorbent assay; GLURP = glutamate-rich protein; MSP = merozoite surface protein; *Pf* = *Plasmodium falciparum*; *Pv* = *P. vivax*.

**Table 2:** Description of frequency of diagnostic test (microscopy and serology) results at individual level (combinations of microscopy and seropositivity by antigen are presented for *Plasmodium falciparum* and *P. vivax* separately)

*P. falciparum* diagnostic tool combinations			
		*Pf*GLURP +	*Pf*GLURP –
Microscopy *Pf* +	*Pf*MSP-1 +	38	5
Microscopy *Pf* +	*Pf*MSP-1 –	3	6
Microscopy *Pf* –	*Pf*MSP-1 +	217	246
Microscopy *Pf* –	*Pf*MSP-1 –	106	4,481
*P. vivax* diagnostic tool combinations			
		*Pv*AMA +	*Pv*AMA –
Microscopy *Pv* +	*Pv*MSP-1 +	4	10
Microscopy *Pv* +	*Pv*MSP-1 –	2	14
Microscopy *Pv* –	*Pv*MSP-1 +	141	381
Microscopy *Pv* –	*Pv*MSP-1 –	78	4,423

AMA = apical membrane antigen; GLURP = glutamate-rich protein; MSP = merozoite surface protein; *Pf* = *P. falciparum*; *Pv* = *P. vivax*.

Data are only presented for individuals with results recorded for *P. falciparum* microscopy, *Pf*GLURP and *Pf*MSP-1 (*N* = 5,102), and individuals with complete results for *P. vivax* microscopy, *Pv*AMA and *Pv*MSP-1 (*N* = 5,053).

**Table 3:** Final Bayesian *Plasmodium falciparum* model developed using data from 62 schools, and *P. vivax* model developed from 71 schools' data (both models retained school-level and spatial random effects)

	*P. falciparum* model parameter value (95% BIC)	*P. vivax* model parameter value (95% BCI)
Altitude	−0.568 (−1.035, −0.087)	–
Slope	−0.595 (−0.996, −0.234)	–
Distance to permanent river	−0.411 (−0.774, −0.036)	–
Population density in 2010	0.418 (−0.107, 0.911)	–
Bare or sparse land (binary)	1.026 (−0.392, 2.298)	–
Urban area (binary)	−3.13 (−6.279, −0.028)	–
*σ*^2^*_school_*	0.254 (0.006, 1.250)	0.288 (0.013, 0.939)
*σ*^2^*_spatial_*	1.183 (0.177, 2.311)	3.631 (1.31, 10.85)
*φ*	9.763 (2.631, 19.03)	0.866 (0.211, 2.093)
Range in kilometer	45.57 (17.54, 127)	548.3 (160.5, 1,592)
DIC	308.8	330.8

BIC = Bayesian information criterion; DIC = deviance information criterion.

School and spatial variance (*σ*^2^*_school_* and *σ*^2^*_spatial_*), rate of decay of spatial correlation (*φ*), range in kilometer at which correlation between schools falls to 5% are presented with 95% Bayesian credible intervals. The *P. falciparum* model includes parameter values and 95% BCI for standardized environmental fixed effects. No environmental fixed effects were retained in the final *P. vivax* model.
