# Supplementary material for: Geostatistical Modeling of Malaria Endemicity using Serological Indicators of Exposure Collected through School Surveys
Source: Am J Trop Med Hyg. 2015 Jul 8;93(1):168–77. doi: 10.4269/ajtmh.14-0620 (PMC4497890; doi:10.4269/ajtmh.14-0620)
Supplement: Supplementary file 1 [file SD7.pdf]

Supplemental Table 1

Univariate frequentist associations of key environmental variables with school seroprevalence of *Plasmodium falciparum* and *P. vivax*

	P. falciparum			*P. vivax*		
	OR	95% CI	*P*	OR	95% CI	*P*
Precipitation						
Annual accumulative	0.978	0.695, 1.374	0.869	1.409	1.047, 1.898	0.024
Annual mean	0.971	0.693, 1.361	0.865	1.400	1.043, 1.880	0.025
Wettest quarter	0.991	0.723, 1.359	0.956	1.479	1.061, 2.062	0.021
Mean at peak transmission	0.781	0.588, 1.039	0.090	0.923	0.711, 1.199	0.549
Annual standard deviation	0.902	0.676, 1.203	0.483	1.336	0.928, 1.924	0.119
Land temperature						
Annual mean	1.272	0.915, 1.767	0.152	0.866	0.657, 1.140	0.305
Mean at peak transmission	1.243	0.892, 1.734	0.199	0.890	0.659, 1.201	0.445
Altitude	0.785	0.590, 1.044	0.096	1.041	0.784, 1.381	0.782
Land gradient	0.565	0.402, 0.796	0.001	0.531	0.336, 0.838	0.007
Distance to						
Any water body	0.808	0.644, 1.014	0.065	0.585	0.422, 0.811	0.001
Permanent water body	0.768	0.609, 0.969	0.026	0.570	0.456, 0.712	< 0.001
Permanent river	0.402	0.172, 0.940	0.036	0.154	0.047, 0.506	0.002
Road	0.983	0.191, 5.057	0.984	1.059	0.206, 5.460	0.945
Land cover type						
Shrubland	1	–	–	1	–	–
Cultivated land	5.320	2.419, 11.70	< 0.001	6.782	2.907, 15.82	< 0.001
Forest	9.5e-7	2.9e-7, 3.1e-6	< 0.001	0.185	0.066, 0.522	0.001
Bare/sparse	4.338	1.979, 9.507	< 0.001	2.236	0.848, 5.900	0.104
Normalized difference vegetation index						
Maximum 2005–2009	0.810	0.658, 0.997	0.047	1.074	0.736, 1.569	0.711
Mean 2005–2009	0.785	0.628, 0.982	0.034	0.903	0.699, 1.166	0.434
Standard deviation 2005–2009	0.824	0.647, 1.049	0.116	1.126	0.770, 1.647	0.541
Maximum 2009	0.846	0.625, 1.146	0.280	1.269	0.831, 1.937	0.271
Mean 2009	0.739	0.571, 0.955	0.021	0.868	0.680, 1.108	0.255
Standard deviation 2009	0.928	0.695, 1.238	0.611	1.398	0.949, 2.060	0.090
Population density						
All ages	0.994	0.720, 1.372	0.970	0.958	0.690, 1.329	0.796
Children < 5 years	0.981	0.696, 1.381	0.912	0.966	0.690, 1.353	0.840
Type of area						
Rural	1	–	–	1	–	–
Peri-urban	0.468	0.182, 1.202	0.115	0.361	0.123, 1.066	0.065
Urban	0.200	0.026, 0.880	0.033	0.065	0.015, 0.283	< 0.001

CI = confidence interval; OR = odds ratio.
